# *Klebsiella pneumoniae* Orbital Cellulitis: Clinical Manifestations and Outcomes in a Tertiary Medical Center in Taiwan

**DOI:** 10.1155/2018/4237573

**Published:** 2018-10-02

**Authors:** Chieh-Hung Yen, Shu-Ya Wu, Yi-Lin Liao

**Affiliations:** ^1^Department of Ophthalmology, Chang Gung Memorial Hospital Linkou Branch, Taoyuan, Taiwan; ^2^College of Medicine, Chang Gung University, Taoyuan, Taiwan; ^3^Department of Ophthalmology, Taipei Tzu Chi Hospital, Buddhist Tzu Chi Medical Foundation, Taipei, Taiwan

## Abstract

**Purpose:**

To report six cases of *Klebsiella pneumoniae* orbital cellulitis without preceding endophthalmitis.

**Method:**

Retrospective chart review.

**Results:**

We reported four females and two males admitted to our hospital for *Klebsiella pneumoniae* orbital cellulitis proven by computed tomographies and bacterial cultures from May 1995 to March 2017. Proptosis, conjunctival congestion, and chemosis and limitation of ocular motility were present in all six patients. Four patients had decreased visual acuities, and three of them recovered completely after treatment. The origin of the infection was sinus in four patients, skin wound in one patient, and sepsis presumably caused by a dental procedure in one patient. Three of all six patients had underlying diabetes mellitus. Two patients had orbital cellulitis before they were diagnosed of diabetes during hospital stay.

**Conclusion:**

Diabetes may be a risk factor of *Klebsiella pneumoniae* orbital cellulitis, especially for those of nonsinus origin.

## 1. Introduction

Orbital cellulitis is an emergency and usually refers to infection spread into the orbital cavity behind the orbital septum. Children are more likely to have this disease than adults. The infections may originate from the adjacent structures such as sinuses (most commonly), periocular skin, lacrimal apparatus, the globe, and oral cavity, or from hematogenous spread. Other causes include direct inoculation by penetrating traumas or surgical procedures and orbital blow-out fractures. If not properly treated, the condition may lead to severe morbidity or even mortality [1–4]. Imaging study, especially computed tomography (CT), has become an important diagnostic tool that can help differentiate preseptal and orbital disease, determining the extent and possible etiology, and choosing the therapeutic strategy [5–7].

The most common microbes causing orbital cellulitis are streptococcus and staphylococcus species. Pathogens in adult patients may be more variable. *Klebsiella pneumoniae* is a Gram-negative bacterium. Infectious diseases caused by *Klebsiella pneumoniae* are more prevalent in Asian countries. It is an important cause of lower-respiratory tract infection in Asia and South Africa, [8] and it is also an important pathogen of endogenous endophthalmitis in East Asian countries. If the clinical course of KP endophthalmitis progresses, orbital involvements will occur [9–11]. However, *Klebsiella pneumoniae* orbital cellulitis with origins other than endophthalmitis is rarely reported [12–15]. Here, we report six cases with this condition.

## 2. Methods

Retrospective chart review was performed on cases with culture-positive *Klebsiella pneumoniae* orbital cellulitis. A total of thirteen cases were identified between May 1995 and March 2017 for detailed review. Seven cases were collected by Dr. Wu and Dr. Liao. Six cases were identified from 113 electronic medical records with the procedural code of orbitotomy with drainage of orbital abscess. Three cases with origins of endophthalmitis were excluded. Another four cases were excluded because of incomplete medical records. Six cases were included in this study.

## 3. Results

The six patients, four females and two males, were all adults with unilateral involvements and had CT-proven orbital cellulitis. The durations between the onset of symptom and the first ophthalmic evaluations by ophthalmologists ranged from 1 to 7 days, with an average of 2.5 days. Symptom of eye pain was documented in four patients. Proptosis, conjunctival congestion, and chemosis and limitation of ocular motility were present in all six patients. Four patients had decreased visual acuities (VA) of various degrees. One patient had intact VA, and one had no available documentation on VA change. Of the four patients with VA impairment, three recovered to normal, and one ended up with hand motion due to compressive optic neuropathy which was more likely to be caused by a previously long-standing fronto-ethmoidal mucocele, rather than orbital cellulitis. Intraocular pressures were available in four patients and were all elevated at initial presentation.

Leukocytosis were seen in all patients, with an average white blood cell (WBC) count of 12.6 × 1000/*µ*L. Five of them had initial white count exceeding 10000/*µ*L. C-reactive protein levels were available in four patients and were elevated in three.

Three patients had underlying diabetes mellitus, and two of them had never been diagnosed of diabetes until the episode of orbital cellulitis. Four patients had an origin of infection from the sinuses. One patient had *Klebsiella pneumoniae* sepsis and meningitis on presentation. One case had a minor trauma on periocular skin and soft tissue without a foreign body.

**Figure 1:**
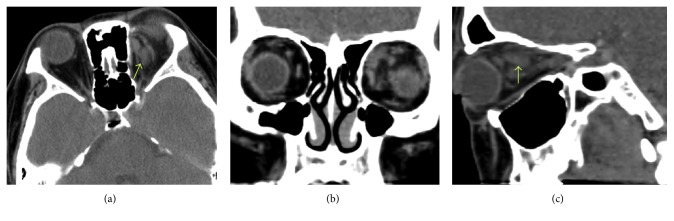
CT scan of case 2. (a) Axial, (b) coronal, and (c) sagittal view. There was retrobulbar infiltration with possible abscess formation (arrows). The sinuses were clear.

**Figure 2:**
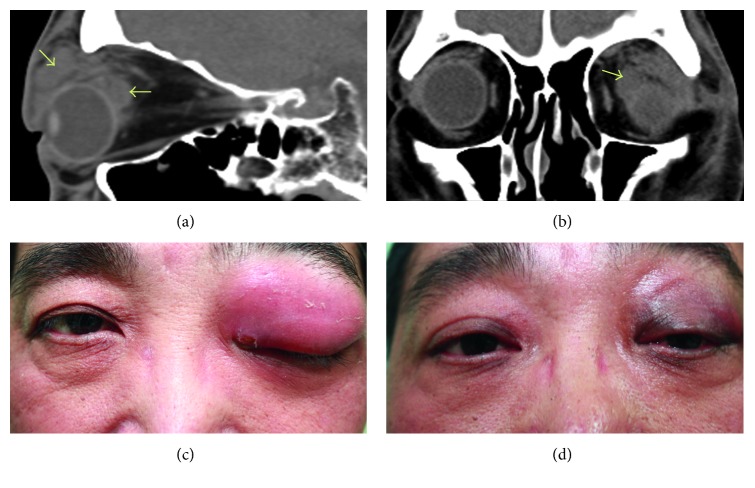
External eye photos and CT scan of case 6. (a, b) CT images. Orbital abscesses seen over the superior orbit and retrobulbar area (arrows). The sinuses were clear. (c) External eye photo before incision and drainage; (d) external photo 1 month after incision and drainage and antibiotic treatment.

Two patients had nonsinusitis origin and true orbital abscesses (not subperiosteal abscesses) (Figures 1 and 2). They were cured by medical treatment. The other four patients with sinusitis had undergone surgical drainage from endonasal approaches and three of them with combined orbitotomy. Average length of stay of the six cases was 15.67 days.

The six cases will be identified by number 1 to 6, according to the date of admission to our hospital, from past to present.  Table 1 lists greater details of the clinical data.

## 4. Discussion

We have found four cases of *Klebsiella pneumoniae* orbital cellulitis in the literature. All four cases were unilateral. In 2001, Lin and Tsai reported a 55-year-old man who had lid abscess caused by sinusitis [12]. However, the authors of this report stated in the discussion section that, judging from the clinical signs and the CT images, they thought it might be a preseptal cellulitis. In 2010, Yang et al. reported a case who had orbital cellulitis with abscess formation associated with cavernous sinus thrombophlebitis. The source of the infection was suspected to be a hematogenous spread from her nasopharyngeal, parapharyngeal, and retropharyngeal abscesses [13]. This case had type 2 diabetes mellitus which had not been diagnosed until the episode of orbital cellulitis. There are also two cases in our series who had undiagnosed diabetes before they had orbital cellulitis. In 2012, Li et al. reported a case with *Klebsiella pneumoniae* orbital cellulitis 9 years after repairment of orbital wall fracture with hydroxyapatite implant [14]. Another case was reported by Murakami et al. in 2016. The patient had underlying type 2 diabetes and had orbital cellulitis and septic emboli in the lungs after tooth extraction. Our case number 2 also had a history of dental procedure prior to the onset of his symptom.  Table 2 summarized the previously reported 3 cases. Lin's case was not included since it should be a preseptal cellulitis case.

*Klebsiella pneumoniae* develops capsules composed of polysaccharides which inhibits phagocytosis of the host and can cause severe invasive diseases. It has been reported that poor glycemic control may stimulate capsular polysaccharide biosynthesis, further impairing the phagocytosis against prominently virulent capsular serotypes K1 and K2 in patients with type 2 diabetes [16–18].

In previous reports, two of the three cases had diabetes. In this series, 50% of the patients had underlying diabetes mellitus. The two without previously recognized diabetes were found to have high HbA1c levels during their hospital stay for orbital cellulitis, and they also had nonsinusitis origin of orbital cellulitis. Diabetes is a known and significant risk factor of *Klebsiella pneumoniae* endogenous endophthalmitis, and it may also be a risk factor of *Klebsiella pneumoniae* orbital cellulitis, especially that without sinusitis as in Cases 2 and 6.

Case 2 had meningitis and orbital cellulitis after a dental procedure. Both conditions were presented upon initial evaluation, and there was no clear temporal relationship between the two. It was hard to tell if one caused the other or the *Klebsiella pneumoniae* bacteremia led to both conditions independently. Moreover, the patient had no evidence of sinusitis and *Klebsiella pneumoniae* is not a common pathogen of odontogenic sinusitis [19–21]. Diabetes may play a role in the pathogenesis of meningitis and orbital cellulitis, through salivary dysfunction, dysbiosis in the oral cavity, interactions between periodontitis and diabetes [22–24], and overall compromised immunity. In our case 2 and R. Murakami's case, oral cavity changes may have caused overgrowth of the bacteria, and compromised immunity facilitated infection after bacteremia caused by dental procedures. Case 6 had an open wound with pus containing *Klebsiella pneumoniae*. Some studies on ocular flora have shown that diabetes may increase the culture rate for Gram-negative bacteria and the proportion of *Klebsiella pneumoniae* isolates [25, 26]. The flora change might also have occurred on the skin of this patient's eyelid.

## 5. Conclusions

Most patients with *Klebsiella pneumoniae* orbital cellulitis have excellent visual outcome after proper antibiotic treatment, with or without surgical treatment. No antibiotic resistance against initial empiric antibiotics was encountered in this series.

Diabetes mellitus may be a risk factor of *Klebsiella pneumoniae* orbital cellulitis. If a patient presents with *Klebsiella pneumoniae* orbital cellulitis and the origin of infection is not from endophthalmitis or sinusitis, an underlying diabetes mellitus should be suspected.

## Figures and Tables

**Table 1:** Clinical data of six patients.

	Age/gender	Laterality	Ophthalmic chief complaint	Initial VA (logMAR)	Final VA (logMAR)	Fever	Diabetes	HbA1c/sugar (AC or random)	Origin of infection	Surgery	WBC (1000/*µ*L)	CRP (mg/L)	Drug resistance test	Hospitalization days
1	69/F	OD	Eye pain, redness, and swelling for 1 day	LP	Hand motion	No	No	–/–	Sinusitis (fronto-ethmoidal mucocele extended to intraconal region, with secondary acute infection)	Functional endoscopic sinus surgery (FESS)	11.5	–	Ampicillin and ticarcillin	20

2	30/M	OS	Headache, eye swelling, and lid drop for 2 days	0.046	0^*∗*^	Yes	Yes	11.9/354 (random)	Sepsis, meningitis (dental procedure prior to the episode)	No	15.7	285.8	Resistance not found	23

3	51/F	OD	Eyelid swelling, redness, and tenderness for 2 days	0.523^*∗*^	0^*∗*^	No	No	–/–	Sinusitis (pyomucocele)	FESS orbitotomy	9.8	10.9	Resistance not found	9

4	71/F	OS	Orbital pain for 1 day	0.222	–	No	Yes	6.1/125 (AC)	Sinusitis (facial bone fractures with an orbital wall defect before the episode)	FESS sequestrectomy orbitotomy	12	–	Resistance not found	17

5	55/F	OS	Eyelid swelling and redness for 2 days	0.301	0	Yes	No	–/114 (random)	Sinusitis	FESS orbitotomy	13.3	131.9	Resistance not found	15

6	48/M	OS	Lid swelling for 7 days^†^	0.824	0^*∗*^	No	Yes	20.7/612 (random)	Trauma	Incision and drainage (at the office)	13.2	3.3	Resistance not found	10

^*∗*^VA with personal glasses. ^†^The patient refused to be admitted when he came the first time; he was admitted 22 days after the initial onset. ^*∗∗*^The data were obtained before admission but after initial treatment, including oral antibiotic and topical antiglaucoma agents. “–”: data not available.

**Table 2:** Summary of previously reported cases.

Case report	Age/gender	Ophthalmic chief complaint	Initial VA (logMAR)	Final VA (logMAR)	Fever	Diabetes	HbA1c	Origin of infection	Surgical drainage
Yang et al. [13]	39/F	Eye pain and swelling for 3 days	– (decreased)	– (reported to have been improved)	Yes	Yes	9.8%	Deep neck infection	Yes
Li et al. [14]	52/M	Eye pain, blepharedema, conjunctival congestion, and proptosis for 1 week	0	– (should be intact)	Yes	–	–	Implant related (orbital wall fracture repair)	Yes
Murakami et al. [15]	49/M	Lid swelling for 5 days	1	−0.079	Yes	Yes	15.3%	Tooth extraction	No

“–”: data not available.

## Data Availability

The clinical data used to support the findings of this study are included within the article.

## Additional Points

*Summary*. We reported six cases of *Klebsiella pneumoniae* orbital cellulitis without preceding endophthalmitis. Five of them had excellent visual outcome. Four cases had orbital infection originated from the sinuses. The other two had previously unrecognized diabetes mellitus which might have contributed to the susceptibility of the host.
